# From hypertransaminasemia to mucopolysaccharidosis IIIA

**DOI:** 10.1186/s13052-014-0097-z

**Published:** 2014-12-02

**Authors:** Paulina Krawiec, Elżbieta Pac-Kożuchowska, Beata Mełges, Agnieszka Mroczkowska-Juchkiewicz, Stanisław Skomra, Agnieszka Pawłowska-Kamieniak, Katarzyna Kominek

**Affiliations:** Department of Paediatrics, Medical University of Lublin, Racławickie 1, 20-059 Lublin, Poland; Department of Paediatrics, Children’s University Hospital in Lublin, Gębali 6, 20-093 Lublin, Poland

**Keywords:** Mucopolysaccharidosis type IIIA, Sanfilippo syndrome, Hypertransaminasemia

## Abstract

**ᅟ:**

Mucopolysaccharidosis type III (MPS III; Sanfilippo syndrome) is a metabolic disorder characterized by the deficiency of a lysosomal enzyme catalyzing the catabolic pathway of heparan sulphate. MPS III presents with progressive mental deterioration, speech delay and behavioural problems with subtle somatic features, which can often lead to misdiagnosis with idiopathic developmental/speech delay, attention deficit/hyperactivity disorder or autism. We report a case of a 5-year-old boy with developmental delay and behaviour problems admitted to the Department of Paediatrics due to chronic hypertransaminasemia. The patient developed normally until the age of 2 years when he was referred to a paediatric neurologist for suspected motor and speech delay. Liver function tests were unexpectedly found elevated at the age of 3.5 years. Physical examination revealed obesity, mildly coarse facial features and stocky hands. He showed mental retardation and mild motor delay. The clinical picture strongly suggested mucopolysaccharidosis. The diagnosis of MPS IIIA was confirmed by decreased activity of heparan N-sulfatase in leucocytes.

**Conclusion:**

We strongly recommend screening for MPS III in children with severe behavioural abnormalities with hyperactivity, psychomotor or speech deterioration and failure to achieve early developmental milestones particularly with facial dysmorphism.

## Background

Mucopolysaccharidosis (MPS) type III, also known as Sanfilippo syndrome, is an autosomal recessive metabolic disorder characterized by the deficiency of a lysosomal enzyme catalyzing the catabolic pathway of glycosaminoglycan (GAG) - heparan sulfate. Based on the deficiency of a specific enzyme in the degradation of heparan sulphate, four different subtypes of MPS III are distinguished, i.e. MPS III A (heparan N-sulfatase), MPS III B (α-N-acetylglucosaminidase), MPS III C (heparan acetyl-CoA: alpha-glucosaminide N-acetyltransferase) and MPS III D (N-acetylglucosamine 6-sulfatase) [1,2].

MPS III presents with progressive mental deterioration, speech delay and behavioural problems with only subtle somatic features, which can often lead to misdiagnosis with idiopathic developmental/speech delay, attention deficit/hyperactivity disorder (ADHD) or autism spectrum disorders [2].

We report a rare case of a boy with unexpected MPS IIIA diagnosis to highlight the importance of paediatric history and physical examination for the diagnostic process and to indicate that patients with metabolic disorders may develop any other chronic diseases.

## Case report

A 5-year-old boy with developmental delay was admitted to the hospital for persistent hypertransaminasemia of 18 months’ duration.

The boy was born at term after the uncomplicated pregnancy by caesarean section due to the ultrasound confirmed large foetus weight and dehiscence of the pubic symphysis. His birth weight was 4,570 g and birth length was 59 cm. At the first minute after birth the Apgar score was 10. No neonatal problems were reported. The boy was the second child in the family and had the older healthy sister. The parents were healthy and non-consanguineous. However, his mother had the history of 2 miscarriages in the first trimester.

According to his parents, the child’s development was normal until the age of 2 years. Head control was achieved by the 3^rd^ month of life, sitting without assistance in the 6^th^ month of life and walking in the 9^th^ month of life. However, he began using single, simple words (i.e. mum, dad) at the age of 18 months. Toilet control was achieved at 3 years of age. Since the 2^nd^ year of life progressive psychomotor developmental delay, restlessness and impulsivity with hyperactivity became apparent, therefore he was referred to a paediatric neurologist. About the age of 5 years lack of speech and toilet control were noticed.

The clinical history revealed recurrent upper respiratory tract infections and recurrent diarrhoea. At present, his stools are rather constipated. He had adenotomy at the age of 2 years. Since birth, the boy had the tendency to excessive weight.

Liver function tests were unexpectedly found to be elevated at the age 3.5 years when he was hospitalized due to pneumonia. Alanine aminotransferase (ALT) was 144 U/L (normal <29 U/L) and aspartate aminotransferase (AST) was 90 U/L (normal <36 U/L). Thereafter, elevated ALT and AST were observed in follow-up laboratory tests.

On admission to our Department his weight was 36.3 kg (> > 97^th^ percentile) and height 115 cm (75^th^ percentile). The Cole’s index was 181.5% and the body mass index was 27.4 kg/m^2^ (> > 97^th^ percentile).

**Figure 1:**
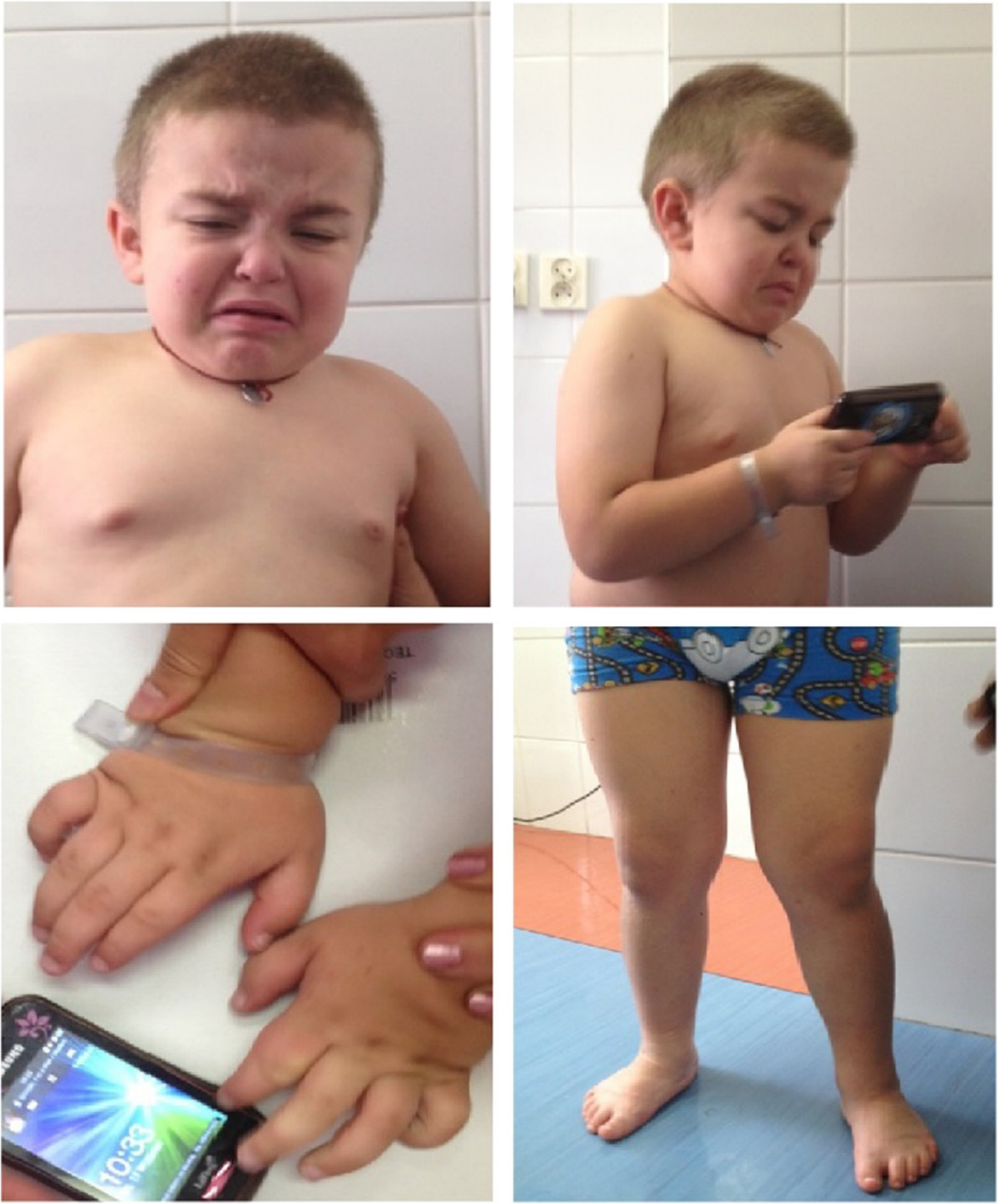
**Dysmorphic features of our patient.** Facial dysmorphism (coarse facial features, the slightly depressed nasal bridge, prominent eyebrows, low set ears, malocclusion, full cheeks, wiry and dry hair and the short neck) and skeletal symptoms (genu valga, varus feet, and stocky hands) seen in our patient.

On physical examination, the boy appeared to have mildly coarse facial features, the slightly depressed nasal bridge, prominent eyebrows, low set ears, malocclusion, full cheeks, wiry and dry hair and the short neck (Figure 1). The boy had excessive lumbosacral lordosis, genu valga (knock-knee deformity) and varus feet. He had broad-base gait with anteversion. His hands were stocky with short fingers. The liver and the spleen were not palpable.

The boy showed mental retardation and mild motor delay. His speech was limited to incomprehensible sounds. The boy presented anxious and restless behaviour.

The clinical picture strongly suggested mucopolysaccharidosis. The daily urinary glycosaminoglycans (GAGs) excretion was 895 mg GAG/g creatinine, i.e. elevated compared to the normal reference level (<88 mg GAG/g creatinine). Elevated GAGs were identified as chondroitin sulphate and heparan sulphate, which suggested mucopolysaccharidosis III. To confirm the diagnosis and to determine the type of MPS the lysosomal enzyme activity was assessed in leucocytes. Heparan N-sulfatase activity was decreased (1.29 nmol/mg protein/18 h) compared to the normal reference range (4.1 ± 1.4 nmol/mg protein/18 h). The activities of other lysosomal enzymes i.e. α-iduronidase, α-N-acetylglucosaminidase, N-acetyl-transferase, glucosamine sulfatase, galactosamine sulfatase, β-glucoronidase, arylsulfatase and iduronide sulfatase were within normal limits. The diagnosis of mucopolysaccharydosis III type A was confirmed.

Moreover, laboratory results revealed hypertransaminasemia with slightly elevated γ-glutamyltranspeptydase. Detailed diagnostic examinations were performed to determine the cause of hypertransaminasemia. Based on the serological tests viral hepatitis caused by hepatitis B virus (HBV), hepatitis C virus (HCV), cytomegalovirus (CMV) and Ebstein – Barr virus (EBV) were excluded. The serum level of creatine kinase, total immunoglobulin G (IgG), α_1_-antitrypsin, lysosomal acid lipase, ceruloplasmin, copper and 24-hour urinary copper were within the normal range. Thus, muscular dystrophy, autoimmune hepatitis, α_1_-antitrypsin deficiency, lysosomal acid lipase deficiency and Wilson’s disease we excluded. Table 1 presents the laboratory results of our patient.

**Table 1:** **Laboratory results of our patient**

**Parameter**	**Result**	**Reference range**
ALT [U/L]	144	<29
AST [U/L]	73	<36
Bilirubin [mg/dL]	1.7	1.5
GGT [U/L]	34	<29
CK [U/L]	98	<149
IgG [mg/dL]	521	504-1464
Total cholesterol [mg/dL]	111	<170
Triglyceride [mg/dL]	58	<75
HBs-Antigen	Negative	Negative
Anti-HCV antibodies	Negative	Negative
Anti-EBV antibodies	Negative	Negative
Anti-CMV antibodies	Negative	Negative
Lysosomal acid lipase [pmol/punch/hr]	0.61	0.37-2.3
α-1-antitrypsin [g/L]	1.3	0.9-2.0
Ceruloplasmin [g/L]	0.3	0.15-0.3
Serum copper [ug/L]	1106	900-1900
24-hour urinary copper [ug/24 h]	<10	<60

On the abdominal ultrasound examination, the bright liver echo pattern was found; the liver and spleen were not enlarged.

Chronic hypertransaminasemia in our patient might be related to mucopolysaccharidosis or result from coexisting non-alcoholic steatohepatitis (NASH). The conclusive diagnostic test should be liver biopsy. However, the child did not undergo liver biopsy in our department as he was referred to the Paediatric Metabolic Diseases and Gastrohepatology Outpatient Clinic.

## Discussion

In MPS III A the deficiency of heparan N-sulfatase results in lysosomal accumulation of undegraded heparan sulphate and eventually in progressive cellular destruction and multisystemic disease [1,2].

Despite biochemical differences, subtypes of MPS III are virtually indistinguishable for clinicians. However, there are data suggesting that the clinical course of MPS IIIA is more severe, with an earlier onset, more rapid progression of symptoms and shorter survival than the other subtypes [1].

The majority of children with MPS IIIA were born after uneventful pregnancies [1,3]. Buhrman et al. found that the most common pregnancy complication was caesarean section due to failure to progress, macrocephaly and breech position, suggesting that the GAGs storage already occur before birth, which was also found in our patient’s perinatal history [4].

Interestingly, Meyer et al. noticed a high number of miscarriages in mothers of children with MPS IIIA (29%). The above data need to be interpreted with caution as the incidence of miscarriages is age-dependent and the exact age of mothers at the time of miscarriage was unknown. However, further research should be performed to investigate the risk of miscarriage in pregnancies involving children with MPS [3].

In general, the early development of a child with MPS IIIA is normal. Nevertheless, the latest studies of the natural course of MPS IIIA showed that the initial symptoms of the disease were present in many patients already during the first year of life [3,4]. Furthermore, Meyer et al. found that three quarters of children with MPS IIIA did not achieve normal early developmental milestones. In 40.6% of children a delay of speech development (late talking >15 months) was observed, while only in 7.2 % delay in motor development (late walking >18 months). In 26.1% of children delay in both speech and motor development was noticed [3]. However, the median age at MPS IIIA diagnosis varied from 3 – 4.5 years [3,4].

Meyer et al. shown that the first symptoms observed by parents in children with MPS were behavioural problems and sleep disturbances [3]. These findings differ from those of Buhrman et al. who found that the most common initial symptom was speech delay (48%), while abnormal behaviours were observed in 9% of patients [4]. In our patient the first symptoms which alarmed his parents were psychomotor progressive retardation and behavioural problems including restlessness, hyperactivity and impulsivity. Sleep disturbances were not observed in our patient.

Severe behavioural and sleep problems are well-established symptoms of Sanfilippo syndrome. The reported behavioural problems include restlessness, severe hyperactivity, impulsivity, temper tantrums, aggression, unusual affect, i.e. laughing or crying fits [2,5]. Sleep disturbances are one of the major concerns in patients with Sanfilippo syndrome, affecting 72 to 96% of patients. The most common sleep problems include settling difficulties, early waking, nocturnal waking, and night-time behaviours like chewing bedclothes, crying, singing or laughing in the night. Some patients show no circadian rhythm [5].

Somatic symptoms in Sanfilippo syndrome are less overt than in the other types of MPS. However, there is a growing body of evidence confirming significant somatic manifestation of MPS IIIA, including coarse facial features, hepatomegaly, hernia, heart disease, hearing impairment, recurrent airway infections, persisting diarrhoea, epilepsy and skeletal abnormalities [3,4,6]. In our patient facial dysmorphism (coarse facial features, the slightly depressed nasal bridge, prominent eyebrows, low set ears, malocclusion, full cheeks, wiry and dry hair and the short neck) and skeletal symptoms (excessive lumbosacral lordosis, genu valga, and varus feet) were evident.

In a child with skeletal abnormality imaging findings may provide a key diagnostic clue. The group of radiological symptoms in mucopolysaccharidosis is known as dysostosis multiplex and include abnormally shaped vertebrae and ribs, enlarged skull, spatulate ribs, hypoplastic epiphyses, thickened diaphyses and bullet-shaped metacarpals. The other common skeletal symptoms are thoracolumbar kyphosis and gibbus deformity, hip dysplasia and genu valgum [7]. Skeletal abnormalities in Sanfilippo syndrome are less prominent than in the other types of MPS and tend to appear after neurocognitive manifestations [4,7]. However, regular imaging of the vertebral column, hips and lower extremities is recommended for patients with MPS [7]. Unfortunately, there is no available X-ray of our patient’s skeletal system.

It should be highlighted that lack of somatic symptoms including facial dysmorphism, should not exclude the diagnosis of MPS IIIA [2]. Large variability in the clinical spectrum of MPS may be a result of residual enzyme activity caused by different gene mutations [3]. However, genotype-phenotype correlations remain unclear and need further investigations [6].

The best to our knowledge chronic hepatitis is not the established symptom of MPS IIIA. Based on detailed diagnostic work-up in our patient, we excluded many possible causes of chronic hypertransaminasemia i.e. infectious hepatitis, muscular dystrophy, autoimmune hepatitis, α_1_-antitrypsin deficiency, lysosomal acid lipase deficiency, and Wilson’s disease. Chronic hypertransaminasemia and the bright liver on ultrasound in our patient may suggest non-alcoholic steatohepatitis (NASH). It may be also related to the liver storage of glycosaminoglycans. However, such an explanation tends to overlook the fact that in another mucopolysaccharidosis – Hunter syndrome (MPS II) – the storage of glycosaminoglycans in the liver does not lead to its dysfunction [8]. Unfortunately, to date no research has been focused on the problem of liver injury in Sanfilippo syndrome. Nevertheless, liver biopsy is required for definitive diagnosis. Thus, we referred the child to the Paediatric Metabolic Diseases and Gastrohepatology Outpatient Clinic, The Children’s Memorial Health Institute in Warsaw, for further diagnostic evaluation and treatment.

The present case underlines the importance of holistic approach to paediatric patient based on thorough medical history and physical examination. It should be noted that early clinical suspicion is crucial to establish the diagnosis.

## Conclusions

We strongly recommend screening for MPS III in children with severe behavioural abnormalities with hyperactivity, psychomotor or speech deterioration and failure to achieve early developmental milestones, particularly with facial dysmorphism. We believe that the present case report will improve awareness of mucopolysaccharidosis in the medical community and result in earlier diagnosis of MPS.

## Consent

Written informed consent was obtained from the patient’s parents for publication of this Case Report and any accompanying images.

## Abbreviations

ADHD: Attention deficit/hyperactivity disorder
ALT: Alanine aminotransferase
AST: Aspartate aminotransferase
CK: Creatine kinase
CMV: Cytomegalovirus
EBV: Ebstein – Barr virus
GAG: Glycosaminoglycan
GGT: γ-glutamyltranspeptydase
HBV: Hepatitis B virus
Hbs: Hepatitis B surface antigen
HCV: Hepatitis C virus
MPS: Mucopolysaccharidosis
NASH: Non-alcoholic steatohepatitis

## References

[1] Valstar MJ, Ruijter GJ, van Diggelen OP, Poorthuis BJ, Wijburg FA (2008). Sanfilippo syndrome: a mini-review. J Inherit Metab Dis.

[2] Wijburg FA, Węgrzyn G, Burton BK, Tylki-Szymańska A (2013). Mucopolysaccharidosis type III (Sanfilippo syndrome) and misdiagnosis of idiopathic developmental delay, attention deficit/hyperactivity disorder or autism spectrum disorder. Acta Paediatr.

[3] Meyer A, Kossow K, Gal A, Mühlhausen C, Ullrich K, Braulke T, Muschol N (2007). Scoring evaluation of the natural course of mucopolysaccharidosis type IIIA (Sanfilippo syndrome type A). Pediatrics.

[4] Buhrman D, Thakkar K, Poe M, Escolar ML (2014). Natural history of Sanfilippo syndrome type A. J Inherit Metab Dis.

[5] Cross EM, Hare DJ (2013). Behavioural phenotypes of the mucopolysaccharide disorders:a systematic literature review of cognitive, motor, social, linguistic and behavioural presentation in the MPS disorders. J Inherit Metab Dis.

[6] Valstar MJ, Neijs S, Bruggenwirth HT, Olmer R, Ruijter GJ, Wevers RA, van Diggelen OP, Poorthuis BJ, Halley DJ, Wijburg FA (2010). Mucopolysaccharidosis type IIIA: clinical spectrum and genotype-phenotype correlations. Ann Neurol.

[7] White KK (2011). Orthopaedic aspects of mucopolysaccharidoses. Rheumatology (Oxford).

[8] Wraith JE, Scarpa M, Beck M, Bodamer OA, De Meirleir L, Guffon N, Meldgaard Lund A, Malm G, Van der Ploeg AT, Zeman J (2008). Mucopolysaccharidosis type II (Hunter syndrome): a clinical review and recommendations for treatment in the era of enzyme replacement therapy. Eur J Pediatr.

